# Supplementary figures and images for: The effect of adjuvants and delivery systems on Th1, Th2, Th17 and Treg cytokine responses in mice immunized with Mycobacterium tuberculosis-specific proteins
Source: PLoS One. 2020 Feb 6;15(2):e0228381. doi: 10.1371/journal.pone.0228381 (PMC7004338; doi:10.1371/journal.pone.0228381)

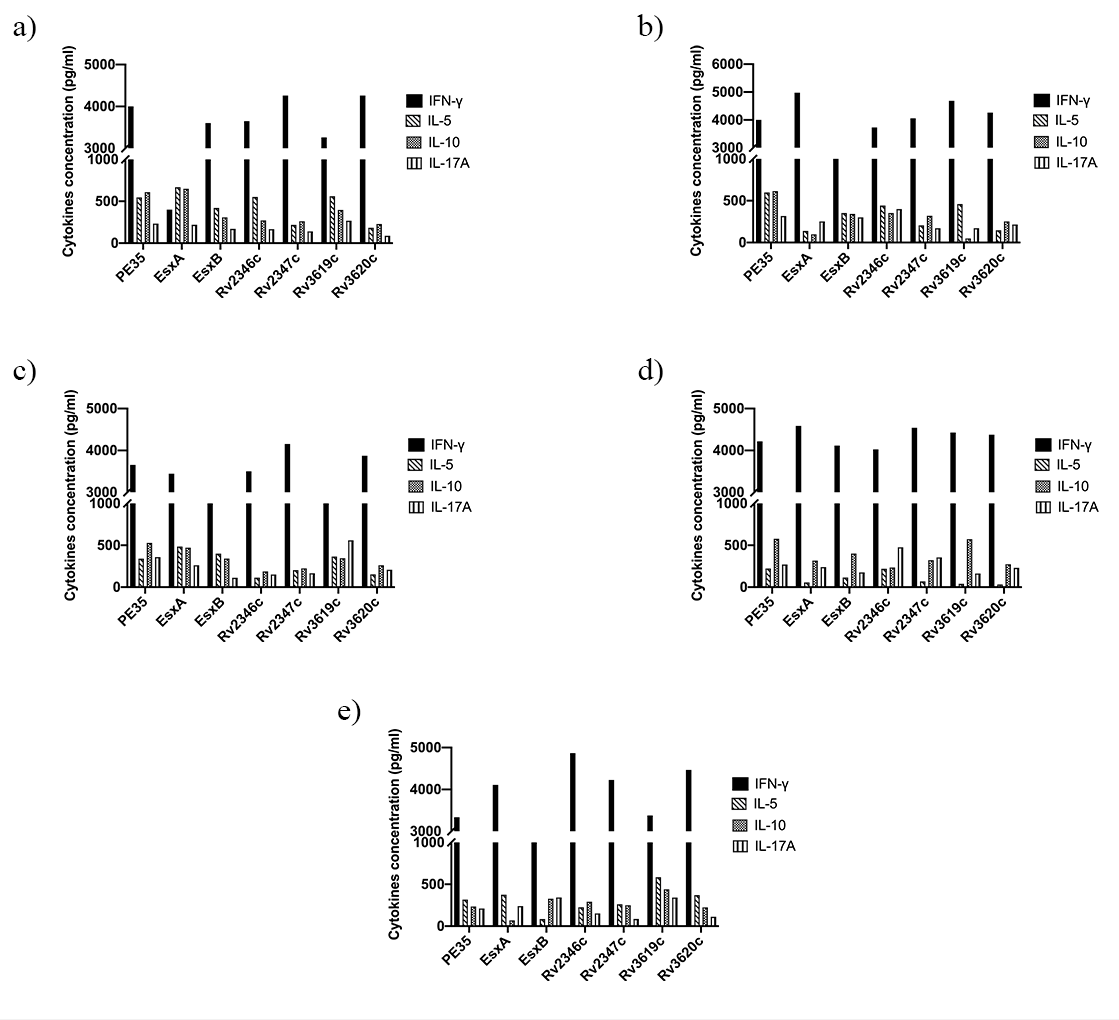

Supplement: S1 Fig — Concentrations (pg/ml) of IFN-ɣ, IL-5, IL-10 and IL-17A in pools of culture supernatants of spleen cells obtained from mice (n = 5 per group) immunized with purified recombinant proteins using a) Alum, b) IFA, c) rM. smegmatis, d) rM. vaccae, and e) DNA vaccine constructs. Spleen cells obtained from immunized mice were cultured in triplicates in the absence of any stimulant (negative control) and in the presence of stimulant (experimental), i.e. Con A. The supernatants were collected on day 3. The culture supernatants from triplicates of negative controls and the stimulant were pooled separately and tested for secreted IFN-γ, IL-5, IL-10 and IL-17A cytokines in duplicate wells of 96-well plates by ELISA. The average values of cytokine concentrations from duplicates are presented in the Figure. (TIF) [file pone.0228381.s001.tif]
