# Supplementary material for: The effect of adjuvants and delivery systems on Th1, Th2, Th17 and Treg cytokine responses in mice immunized with Mycobacterium tuberculosis-specific proteins
Source: PLoS One. 2020 Feb 6;15(2):e0228381. doi: 10.1371/journal.pone.0228381 (PMC7004338; doi:10.1371/journal.pone.0228381)
Supplement: Attachment — Submitted filename: Response to Reviewers.docx [file pone.0228381.s003.docx]

Response to reviewer’s comments

Reviewer #1:

Comment: According to the comments, the manuscript was properly revised with improvement. I have one minor comment on statistics. Please clarify the objectives of comparison in all figures.

Response: We would like to thank the reviewer for stating that the manuscript was properly revised with improvement.

The objective of comparison in all figures was to determine which immunization followed by stimulation of spleen cells in vitro resulted in secretion of significant concentrations of the individual cytokines. This information has been added in the materials and methods under data analysis.

Reviewer #2: -

Comment: The authors have addressed some comments but they still need to make it clearer in their discussion how measuring only immune responses rather than protection is a limitation due to lack of immune correlates in TB.

Response: We thank the referee for stating that the authors have addressed some comments. Furthermore, as per the suggestion of the reviewer, we have added in the end of discussion how measuring only immune responses rather than protection is a limitation due to lack of immune correlates in TB.

Comment: Affinity columns will not remove endotoxin for the protein preparations. The authors should indicate at least the approximate levels of LPS. Big differences in endotoxin levels between proteins should be noted.

Response: We had not measured the concentration of endotoxin in our recombinant protein preparations and hence it is not possible to indicate the approximate levels of LPS. However, we have used purified recombinant proteins only for immunizations using IFA and Alum as adjuvants. Other immunizations using recombinant mycobacteria (r *M. vaccae* and r*M. smegmatis*) and rDNA vaccine constructs will not have a problem of LPS contamination. The endotoxin contamination would have been a major concern in our study if we had used the recombinant proteins for in vitro stimulation of spleen cells for cytokine secretions. However, to avoid the non-specific stimulation of spleen cells due to the endotoxin contamination of the recombinant proteins, we have used chemically synthesized peptides covering the sequences of the individual proteins, as stated in the materials and methods.

Comment: Thanks to the authors for including antibody data. Can the authors clarify whether the serum was collected from the one experiment or whether sera from different experiments were used to create Figure 8?

Response: The serum samples were collected from the one experiment to create Figure 8 (Figure 7 in the revised manuscript).

Comment: The comment about the use of BALB/c mice and potential Th bias has not been adequately addressed.

Response: We agree with the comment of the reviewer that the use of BALB/c mice and the potential Th bias was not adequately addressed. The reason being that the experimental studies have shown divergent results on the Th responses in response to mycobacterial antigens in mice of different genetic backgrounds as given below.

A. Examples of studies showing that C57BL/6 mice have Th1 profile and BALB/c mice have Th2.
1. Huygen K, Abramowicz D, Vandenbussche P, Jacobs F, De Bruyn J, Kentos A, Drowart A, Van Vooren JP, Goldman M. Spleen cell cytokine secretion in *Mycobacterium bovis* BCG-infected mice. Infect Immun. 1992 Jul;60(7):2880-6.
2. Wakeham J, Wang J, Xing Z. Genetically determined disparate innate and adaptive cell-mediated immune responses to pulmonary *Mycobacterium bovis* BCG infection in C57BL/6 and BALB/c mice.

Infect Immun. 2000 Dec;68(12):6946-53.

3. Paula MO, Fonseca DM, Wowk PF, Gembre AF, Fedatto PF, Sérgio CA, Silva CL, Bonato VL.Host genetic background affects regulatory T-cell activity that influences the magnitude of cellular immune response against *Mycobacterium tuberculosis*. Immunol Cell Biol. 2011 May;89(4):526-34.
4. Sérgio CA, Bertolini TB, Gembre AF, Prado RQ, Bonato VL. CD11c(+)  CD103(+) cells of *Mycobacterium tuberculosis*-infected C57BL/6 but not of BALB/c mice induce a high frequency of interferon-γ- or interleukin-17-producing CD4(+) cells.

Immunology. 2015 Apr;144(4):574-86.

B. Examples of studies showing that BALB/c mice have a stronger Th1 and Th17 responses than C57BL/6 mice or both mouse strains have similar Th profiles.
1. Garcia-Pelayo MC, Bachy VS, Kaveh DA, Hogarth PJ. BALB/c mice display more enhanced BCG vaccine induced Th1 and Th17 response than C57BL/6 mice but have equivalent protection. Tuberculosis (Edinb). 2015;95(1):48-53.
2. Leung-Theung-Long S, Gouanvic M, Coupet CA, Ray A, Tupin E, Silvestre N, Marchand JB, Schmitt D, Hoffmann C, Klein M, Seegren P, Huaman MC, Cristillo AD, Inchauspé G. A Novel MVA-Based Multiphasic Vaccine for Prevention or Treatment of tuberculosis induces broad and multifunctional cell-mediated immunity in mice and primates. PLoS One. 2015;10(11):e0143552.
3. Yadav B, Malonia SK, Majumdar SS, Gupta P, Wadhwa N, Badhwar A, Gupta UD, Katoch VM, Chattopadhyay S. Constitutive expression of SMAR1 confers susceptibility to *Mycobacterium tuberculosis* infection in a transgenic mouse model. Indian J Med Res. 2015 Dec;142(6):732-41.
4. Walton CB, Inos AB, Andres OA, Jube S, de Couet HG, Douglas JT, Patek PQ, Borthakur D. Immunization with hybrid recombinant *Mycobacterium tuberculosis* H37Rv proteins increases the TH1 cytokine response in mice following a pulmonary instillation of irradiated mycobacteria. Vaccine. 2008;26(34):4396-402.
5. Roupie V, Romano M, Zhang L, Korf H, Lin MY, Franken KL, Ottenhoff TH, Klein MR, Huygen K. Immunogenicity of eight dormancy regulon-encoded proteins of *Mycobacterium tuberculosis* in DNA-vaccinated and tuberculosis-infected mice. Infect Immun. 2007;75(2):941-9.

Hence, selection of BALB/c mice based on a specific Th profile does not seem to be justified and, therefore, we did not address this issue.

Comment: Figure 6: It is not entirely clear what the data presented are. Have the authors pooled all the data for the same protein from different experiments? If this is the case, how do they account for the huge differences in the non-stimulated controls between experiments? I would remove this figure as it is not scientifically accurate to pool responses from non-direct replicate experiments into one bar.

Response: Figure 6: Yes, all the data were pooled for the same protein from different experiments. We agree with the reviewer and Figure 6 has been removed in the revised manuscript.
